# Case Report: Fatal recurrent rupture of multiple pancreatic pseudoaneurysms following acute alcoholic pancreatitis

**DOI:** 10.3389/fmed.2026.1948368

**Published:** 2026-10-08

**Authors:** Xin Wang, Deyu Zhang, Dongyang Wang, Hao Lin, Wenxiu Zhang, Xingshun Qi

**Affiliations:** Department of Gastroenterology, General Hospital of Northern Theater Command, Shenyang, China

**Keywords:** acute alcoholic pancreatitis, gastrointestinal hemorrhage, hemorrhagic shock, pancreatic pseudoaneurysm rupture, transarterial embolization

## Abstract

**Background:**

Pancreatic pseudoaneurysm (PPA) is a severe and life-threatening vascular complication of pancreatitis. This case highlights the critical need for comprehensive vascular assessment and surveillance in patients with alcoholic pancreatitis to prevent fatal adverse outcomes.

**Case presentation:**

A 53-year-old man with a 30-year history of heavy alcohol intake (500 mL of 52% ABV Baijiu daily, corresponding to an estimated 205 g pure ethanol per day) presented to our emergency department with hematemesis and hemorrhagic shock on 2 April 2026. He was diagnosed with acute pancreatitis complicated by a pancreatic pseudocyst at another hospital in November 2025 but did not receive standardized follow-up and treatment. Contrast-enhanced CT confirmed rupture of a pancreatic head pseudoaneurysm with hemorrhage extending into the duodenum. Emergency transarterial embolization of the gastroduodenal artery was performed, and hematemesis resolved temporarily. Subsequently, the patient developed progressive abdominal pain, distension, and peritoneal irritation signs. Repeat CT confirmed recurrent intra-abdominal hemorrhage. A second digital subtraction angiography (DSA) identified an unrecognized feeding branch arising from the superior mesenteric artery (SMA) supplying the pancreatic head pseudoaneurysm, which was successfully embolized. The patient's condition stabilized temporarily, with a gradual increase in hemoglobin levels. On 14 April 2026, the patient suffered sudden severe abdominal pain and abdominal distension. Follow-up contrast-enhanced CT revealed enlargement and rupture of a pre-existing tiny pseudoaneurysm in the splenic hilum, resulting in massive intra-abdominal hemorrhage. The patient developed refractory hemorrhagic shock followed by cardiac arrest during preparation for emergency endovascular intervention and ultimately died. Post-embolization hemodynamic changes may have contributed to progressive enlargement and subsequent rupture of the pre-existing lesion, although this causal mechanism cannot be definitively proven in this single case.

**Conclusion:**

This case illustrates that pancreatitis-associated pseudoaneurysms may be multifocal. Successful control of a dominant bleeding lesion does not eliminate the risk of rupture of the separate pre-existing small pseudoaneurysm, and incomplete initial angiography limited only to the celiac trunk may miss SMA feeding branches, even after technically successful initial endovascular intervention for pancreatitis-associated pseudoaneurysm.

## 1 Introduction

Pancreatic pseudoaneurysm (PPA) is a severe and life-threatening vascular complication of pancreatitis that can occur in both acute and chronic forms of the disease (1, 2). Long-term excessive alcohol abuse is one of the major risk factors for pancreatic pseudoaneurysm formation (3). Activated pancreatic proteolytic enzymes erode the walls of peripancreatic arteries, resulting in pseudoaneurysm formation within pancreatic pseudocysts or adjacent inflammatory tissues (4). Unlike true arterial aneurysms, PPAs lack an intact three-layer vascular structure and are only confined by fragile inflammatory fibrous tissue, rendering them highly susceptible to spontaneous rupture (5).

Ruptured PPAs typically present with massive gastrointestinal hemorrhage or intra-abdominal hemorrhage, which may rapidly progress to life-threatening hemorrhagic shock, with an overall mortality rate exceeding 50% (6). Transarterial embolization has emerged as the first-line minimally invasive treatment for ruptured PPAs, achieving high immediate technical success (7). However, multiple feeding vessels, incomplete embolization, and hemodynamic redistribution after intervention may contribute to recurrent bleeding and progressive vascular lesions, which remain major clinical challenges (8). Recurrent hemorrhage following initial intervention is associated with significantly increased mortality (9).

We herein report a fatal case of recurrent rupture of multiple pancreatic pseudoaneurysms in a patient with long-term heavy alcohol consumption and acute alcoholic pancreatitis, aiming to highlight the clinical risks, management challenges and preventive strategies related to this highly lethal complication.

## 2 Case presentation

### 2.1 Patient history

A 53-year-old male presented to the emergency department on 2 April 2026 with a 6-hour history of acute hematemesis and severe abdominal distension. The patient had a 30-year history of heavy alcohol consumption, with a daily intake of approximately 500 mL of 52% ABV baijiu, equivalent to an estimated 205 g of pure ethanol per day. In November 2025, he was admitted to a local hospital with abdominal pain and distension and was diagnosed with acute pancreatitis complicated by a pancreatic pseudocyst. He declined standardized regular treatment and continued heavy alcohol consumption after discharge. He had no history of hypertension, diabetes mellitus, liver cirrhosis, or previous abdominal surgery.

### 2.2 Admission physical examination and laboratory findings

On initial admission, the patient presented with unstable vital signs: blood pressure 70/40 mmHg, heart rate 135 beats per minute, respiratory rate 26 breaths per minute, and body temperature 36.8 °C. Physical examination revealed pale conjunctiva, cold extremities, marked abdominal distension, generalized abdominal tenderness, rebound tenderness, and diminished bowel sounds.

Baseline laboratory tests indicated severe anemia and significant systemic stress: hemoglobin 52 g/L (reference range: 130–175 g/L), white blood cell count 18.6 × 10⁹/L (4.0–10.0 × 10⁹/L), and platelet count 210 × 10⁹/L (100–300 × 10⁹/L). Serum amylase was markedly elevated at 480 U/L (30–110 U/L). Renal function parameters were also elevated: blood urea nitrogen 12.5 mmol/L (3.2–7.1 mmol/L) and serum creatinine 138 μmol/L (57–111 μmol/L). Based on the clinical manifestations and laboratory results, a preliminary diagnosis of ruptured pancreatic pseudoaneurysm with secondary duodenal hemorrhage and hemorrhagic shock was established.

### 2.3 Imaging findings and interventional management

#### 2.3.1 Initial contrast-enhanced computed tomography (CT)

**E**mergency contrast-enhanced abdominal CT detected a large pancreatic head pseudoaneurysm with active contrast extravasation into the duodenum and peripancreatic hematoma, confirming ruptured pancreatic head pseudoaneurysm causing duodenal hemorrhage (Figure 1). Based on the patient's history, physical examination and laboratory findings, the presentation was considered to represent a delayed vascular complication related to the previous episode and a residual pseudocyst.

**Figure 1:**
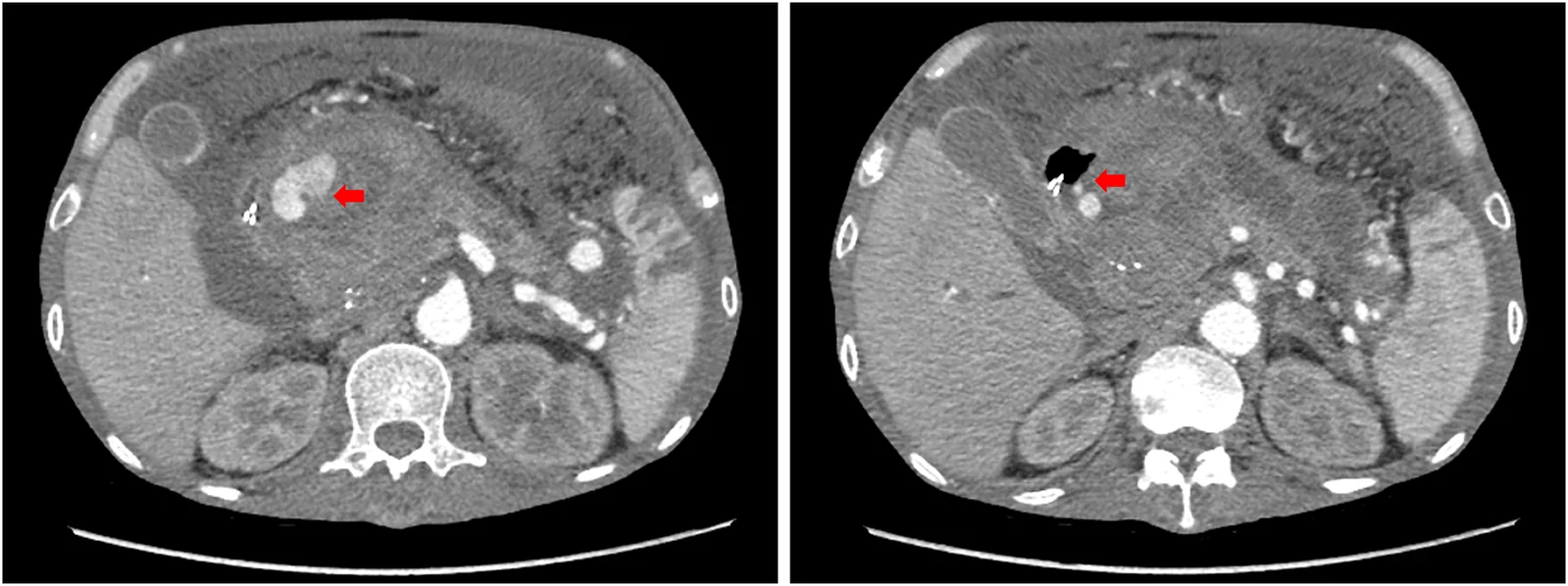
Arterial-phase contrast-enhanced abdominal CT demonstrating a pancreatic head pseudoaneurysm (red arrow) with active contrast extravasation into the duodenum and associated peripancreatic hematoma.

#### 2.3.2 First emergency DSA and transarterial embolization (TAE)

Emergency DSA confirmed a bleeding pseudoaneurysm originating from the gastroduodenal artery. Immediate therapeutic TAE was performed using gelatin sponge particles and metallic coils to achieve occlusion of the feeding artery (Figure 2). Given the patient's critical hemorrhagic shock and severe intraoperative agitation, only selective angiography of the celiac trunk was performed. Active contrast extravasation was identified from a pseudoaneurysm arising from the gastroduodenal artery. Targeted segmental embolization was performed using detachable microcoils combined with gelatin-sponge particles. Post-embolization angiography demonstrated complete occlusion of the celiac-origin bleeding vessel with no residual contrast filling within the visualized portion of the pseudoaneurysm, achieving immediate technical hemostasis for the identified bleeding source. During the emergency DSA procedure, the patient was agitated and had poor cooperation, which precluded comprehensive angiographic assessment. Only the celiac axis was selectively evaluated at that time, but the superior mesenteric artery was not interrogated.

**Figure 2:**
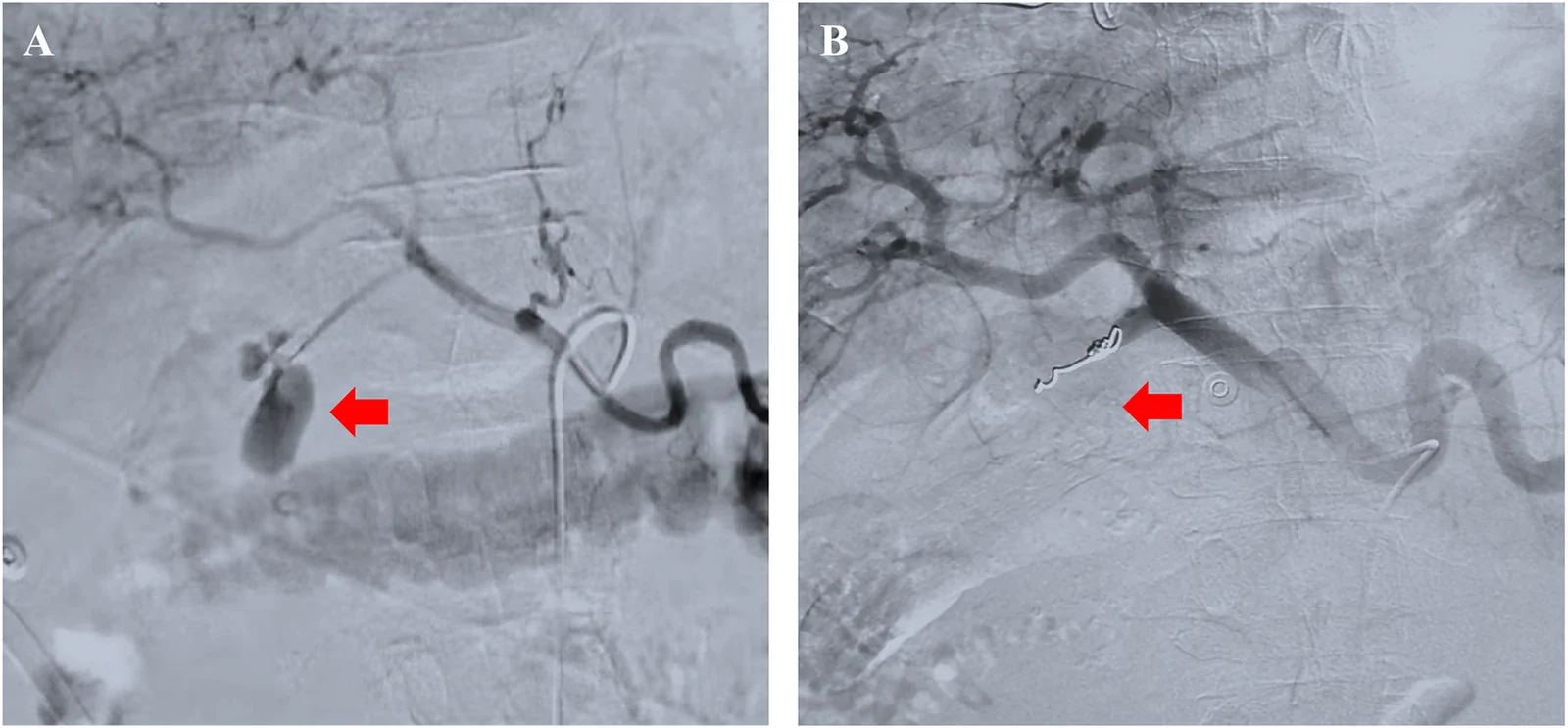
**(A)** Pre-embolization digital subtraction angiography demonstrating a gastroduodenal artery pseudoaneurysm (red arrow) with active contrast extravasation indicative of ongoing bleeding. **(B)** Post-embolization angiography demonstrated no residual filling from the celiac-side angiographic injection (red arrow), indicating immediate hemostasis of the identified bleeding source.

Following the initial embolization, acute hematemesis resolved completely. A repeat upper gastrointestinal endoscopy performed 24 h post-procedure showed no evidence of active bleeding in the esophagus, stomach, or duodenum. However, 48 h after the first intervention, the patient developed worsening abdominal pain and distension with persistent peritoneal irritation signs. Follow-up non-contrast abdominal CT revealed new high-density free intra-abdominal fluid, consistent with acute recurrent intra-abdominal hemorrhage (Figure 3).

**Figure 3:**
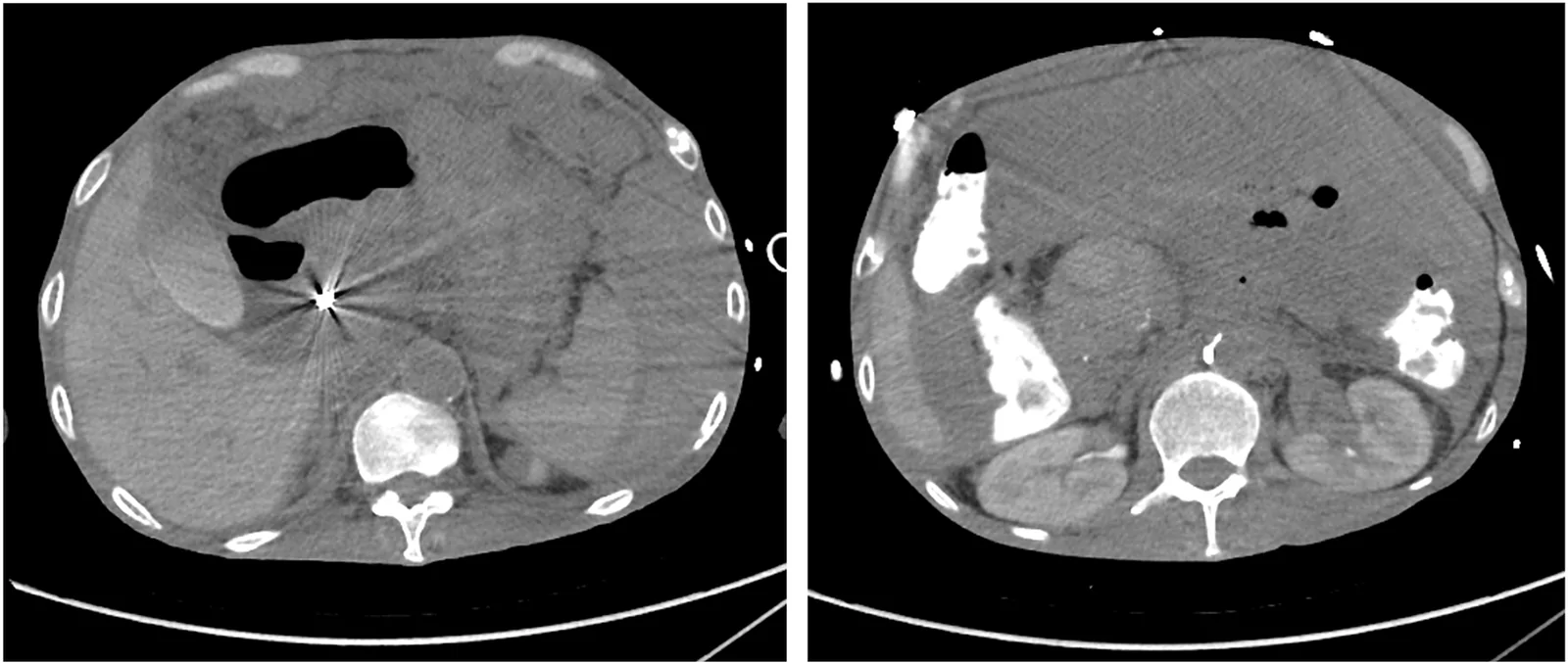
Non-contrast abdominal CT obtained 48 h after initial embolization demonstrating new high-density free intra-abdominal fluid consistent with acute intra-abdominal hemorrhage.

#### 2.3.3 Second emergency DSA and supplementary embolization

After recurrent intra-abdominal hemorrhage, repeated DSA was performed with full evaluation of both the celiac axis and superior mesenteric artery. Angiography identified an unrecognized feeding branch from the SMA supplying the residual pancreatic head pseudoaneurysm. Supplemental embolization of this collateral feeding vessel was performed using detachable microcoils together with gelatin-sponge particles. Final angiography confirmed complete exclusion of the pseudoaneurysm sac without residual perfusion or collateral blush (Figure 4).

**Figure 4:**
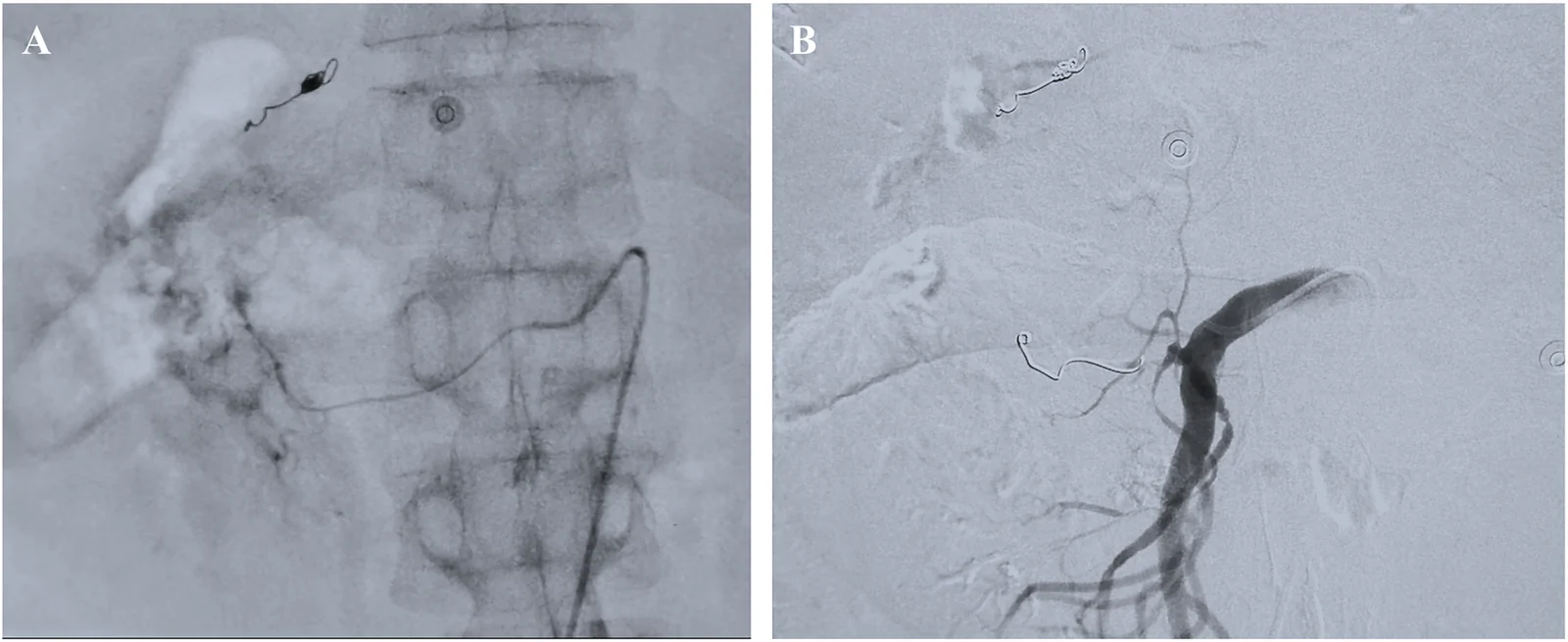
**(A)** Second digital subtraction angiography demonstrating an additional feeding branch from the superior mesenteric artery (SMA) supplying the pancreatic head pseudoaneurysm before supplementary embolization. **(B)** Post-supplementary embolization digital subtraction angiography demonstrating occlusion of the pancreatic head pseudoaneurysm with no residual contrast extravasation.

#### 2.3.4 Post-interventional clinical course and outcome

After the second embolization, the patient's vital signs gradually stabilized. Under comprehensive supportive management including fluid resuscitation, blood transfusion, acid suppression and symptomatic care, the patient's hemoglobin increased progressively from 68 g/L to 95 g/L within 9 days, alongside gradual resolution of abdominal pain and signs of peritoneal irritation.

On 14 April 2026 (10 days following the second embolization), the patient suffered sudden recurrence of severe abdominal pain and distension, accompanied by an acute drop in blood pressure. Emergency contrast-enhanced CT demonstrated marked shrinkage of the original pancreatic-head pseudoaneurysm. Notably, a small pseudoaneurysm initially observed in the splenic hilum on the admission CT exhibited significant enlargement (up to 2.5 × 2.0 cm), accompanied by massive acute intra-abdominal hemorrhage **(**Figure 5**)**. The lesion was already present at the initial presentation but remained small and clinically stable. Dramatic hemodynamic redistribution after repeated transarterial embolization could theoretically have increased regional arterial perfusion pressure, which cannot be excluded as a factor leading to progressive dilatation, wall injury, and eventual rupture of the pre-existing minor pseudoaneurysm.

**Figure 5:**
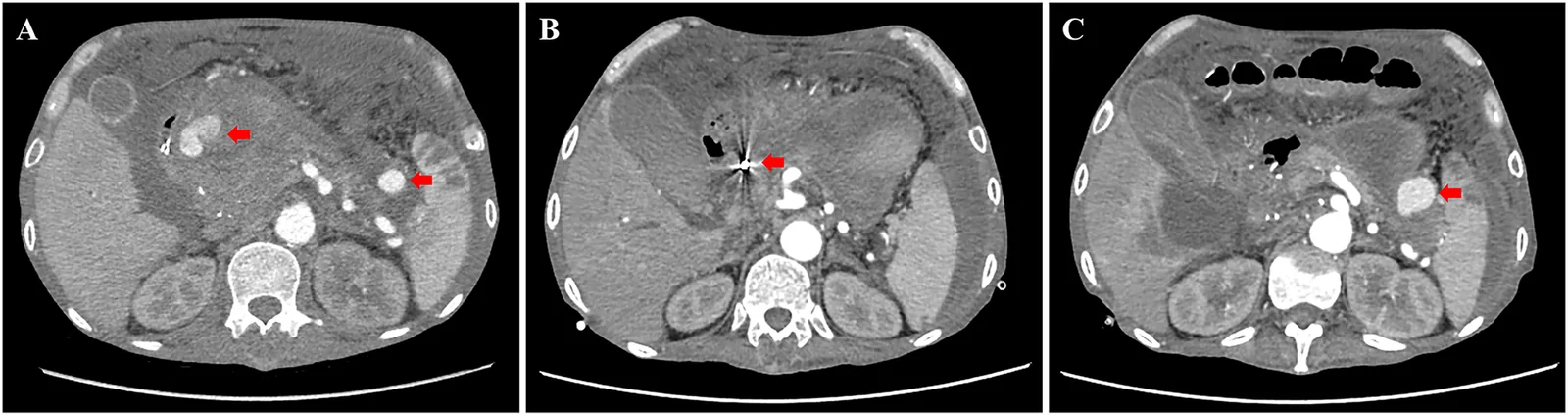
Serial axial arterial-phase contrast-enhanced abdominal CT demonstrating the evolution of multiple pseudoaneurysms. **(A)** Pre-embolization CT obtained on April 2, 2026 demonstrated a pseudoaneurysm of the pancreatic head (left red arrows) arising from a branch of the gastroduodenal artery, as well as a small pre-existing pseudoaneurysm (0.7 × 1.0 cm) at the splenic hilum (right red arrows) originating from a splenic arterial branch. **(B)** Post-embolization CT obtained on April 14, 2026 showed embolization coils (red arrows) within the pancreatic-head region, with complete obliteration of the pancreatic-head pseudoaneurysm. **(C)** CT acquired on April 14, 2026 demonstrated marked interval enlargement of the splenic-hilum pseudoaneurysm (red arrows) to 2.5 × 2.0 cm with peri-lesional hemorrhagic signs.

The patient was prepared urgently for repeat emergency endovascular intervention. While being transferred to the angiography suite, he developed refractory hemorrhagic shock followed by cardiac arrest. Immediate standard cardiopulmonary resuscitation was performed, but return of spontaneous circulation could not be achieved. The patient was subsequently dead. Autopsy was not performed because the patient's next of kin refused consent.

## 3 Discussion

This fatal case highlights that acute pancreatitis may produce multifocal peripancreatic pseudoaneurysms. Even after successful hemostasis of the dominant bleeding lesion, the small pre-existing pseudoaneurysm may enlarge and rupture. A core clinical lesson is that urgent angiography limited only to the celiac trunk may overlook SMA feeding branches; comprehensive evaluation of both celiac and SMA circulations should be performed whenever the patient's hemodynamic status permits. Hemorrhagic stabilization after transarterial embolization (TAE) does not eliminate bleeding risk from other separate vascular lesions in the setting of ongoing pancreatic inflammation. Initial embolization may achieve immediate technical hemostasis of a visualized bleeding vessel, but it does not guarantee full exclusion of the pseudoaneurysm if other feeding arteries remain unassessed. This discussion addresses inflammatory pathogenesis, imaging findings, therapeutic limitations and individualized surveillance for this vascular emergency.

Acute pancreatitis may cause local tissue injury and peripancreatic vascular damage. Released pancreatic enzymes including trypsin and elastase could erode peripancreatic arterial walls. Under conditions of acute exudation, edema and pseudocyst formation, arteries may suffer enzymatic digestion and mechanical compression, which might lead to arterial wall weakness and hematoma formation (10). Encapsulated hematomas can form pseudoaneurysms, most frequently involving the gastroduodenal artery, splenic artery and SMA branches (11).

Long-term alcohol abuse was the underlying predisposing factor in this patient. A tiny pseudoaneurysm in the splenic hilum was already visible on admission CT. Persistent active inflammation may weaken peripancreatic arterial walls and could be one plausible pathological contributor to lesion progression. Repeated embolization might alter regional hemodynamics, possibly raising perfusion pressure in remaining peripancreatic branches. Fragile pre-existing pseudoaneurysms may not tolerate elevated pressure, which could facilitate rapid dilation and rupture (12). Several competing explanations may account for fatal re-bleeding on 10 days after the second embolization: pre-existing small pseudoaneurysm, post-embolization hemodynamic redistribution, pancreatitis-related autodigestive vascular injury, or rare vessel recanalization. Definitive proof of any single mechanism cannot be obtained from this single case.

Contrast-enhanced CT is the first-line modality for detecting pancreatitis-associated vascular complications, with high diagnostic sensitivity (13). It evaluates pancreatic necrosis, peripancreatic collections, pseudocysts and pseudoaneurysms to guide emergency management. DSA remains the gold-standard for vascular mapping and TAE guidance (14). In this case, initial CT identified the ruptured pancreatic-head pseudoaneurysm but missed an additional SMA-derived feeding branch. Acute-pancreatitis-driven vascular remodeling may produce multi-vessel supply; complete angiography of both the celiac trunk and SMA is required for complex pseudoaneurysms to avoid missed feeding vessels and early re-bleeding.

A previous report demonstrated that TAE achieved a high rate of immediate technical success in 11 of 13 patients with ruptured pseudoaneurysms (85%) (15). Nevertheless, persistent acute inflammation may be one important risk factor for re-bleeding (16). Unlike traumatic pseudoaneurysms, pancreatitis-related vascular lesions may progress while inflammation persists. Even after hemostasis, ongoing enzymatic injury might further weaken arterial walls. Pre-existing small pseudoaneurysms may dilate rapidly within 10 days post-embolization and lead to fatal hemorrhagic shock before salvage intervention. Open surgery is reserved for refractory bleeding after TAE failure but carries high peri-operative mortality due to inflammation, coagulopathy and organ dysfunction (17). This case illustrates potential limitations of embolization alone in uncontrolled acute pancreatitis.

Early comprehensive management of acute pancreatitis including fluid resuscitation, inflammation control and alcohol abstinence may limit progressive vascular injury (18–21). Such early interventions could also mitigate endothelial dysfunction and vascular damage, thereby potentially limiting progressive vascular complications and improving overall clinical outcomes (22, 23). Timely drainage of peripancreatic collections may reduce arterial compression (24). In retrospective series, 1-3-month post-embolization CT imaging is sometimes used for patients whose inflammation has resolved (25). This is not a guideline-recommended interval and may be inappropriate for patients with ongoing active pancreatitis. As shown here, life-threatening hemorrhage may occur as early as 10 days after embolization in high-risk individuals. Individualized short-interval imaging and close clinical monitoring should therefore be considered. Conclusions from a single case cannot be generalized, but this report provides a practical clinical warning. Multidisciplinary collaboration between emergency, gastroenterology, interventional radiology and general surgery may improve outcomes for these high-risk patients (26). Since the patient died from fatal intra-abdominal haemorrhage, patient-reported perspectives could not be obtained. A thorough review of the patient's medical history, physical examination and available imaging revealed no signs of arterial dissection, aneurysms, skin hyperelasticity joint hypermobility or a family history of connective tissue diseases; however, a definitive exclusion of an underlying connective tissue disorder could not be established in the absence of autopsy.

## 4 Conclusion

Pancreatic pseudoaneurysm rupture represents a life-threatening vascular complication following alcohol-associated acute pancreatitis. This case illustrates that pancreatitis-associated pseudoaneurysms may be multifocal; control of the dominant bleeding source does not eliminate the risk of subsequent rupture of separate pre-existing small pseudoaneurysms. Awareness of multifocal vascular lesions and careful reassessment after initial hemostasis is critical. Although TAE can successfully control initial hemorrhage, persistent pancreatic inflammation and post-embolization hemodynamic changes may have contributed to the rapid dilation and rupture of pre-existing tiny pseudoaneurysms, leading to fatal recurrent bleeding. To reduce the mortality of this severe complication, clinicians should prioritize early and standardized management of acute pancreatitis and pseudocysts, enforce strict alcohol abstinence, perform comprehensive angiographic evaluation whenever feasible during intervention, and adopt individualized short-interval imaging surveillance for high-risk patients. In addition, in the context of acute alcoholic pancreatitis, awareness of multifocal vascular lesions and careful reassessment after initial hemostasis are of great importance for prognosis and management. Findings from this single case cannot be generalized, but provide a meaningful clinical warning for the management of pancreatitis-associated vascular emergencies.

## Data Availability

The original contributions presented in the study are included in the article/Supplementary Material, further inquiries can be directed to the corresponding author.
