# Supplementary material for: Case Report: Fatal recurrent rupture of multiple pancreatic pseudoaneurysms following acute alcoholic pancreatitis
Source: Front Med (Lausanne). 2026 Oct 8;13:1948368. doi: 10.3389/fmed.2026.1948368 (PMC13689719; doi:10.3389/fmed.2026.1948368)
Supplement: Supplementary file 1 [file Table1.docx]

**Case Report: Fatal recurrent rupture of multiple pancreatic pseudoaneurysms following acute alcoholic pancreatitis**

Xin Wang^1#^, Deyu Zhang^1#^, Dongyang Wang^1^, Hao Lin^1^, Wenxiu Zhang^1^, Xingshun Qi^1^*

^1^Department of Gastroenterology, General Hospital of Northern Theater Command, 110840 Shenyang, China.

^#^These authors contributed equally to this work and share first authorship.

*Corresponding author: Xingshun Qi, Email: xingshunqi@126.com

Supplemental Table 1. Timeline of clinical presentation and management for the index case

| **Timeline** | **Clinical manifestations** | **Laboratory findings** | **Imaging**  **findings** | **Medical management** | **Outcome** |
| --- | --- | --- | --- | --- | --- |
| November 2025 | Epigastric discomfort, history of alcohol‑induced acute pancreatitis | Laboratory data unavailable | Abdominal ultrasound demonstrated pancreatic pseudocyst formation; other detailed imaging data missing | Conservative supportive treatment in hospital | Partial improvement of abdominal symptoms |
| 2 April 2026 | Acute hematemesis, severe abdominal distension and hemodynamic instability. Vital signs: heart rate 135 beats/min, blood pressure 70/40 mmHg. | Hemoglobin 52 g/L, lactate 10.1 mmol/L, CRP 37 mg/L, procalcitonin 0.16 ng/ml. Coagulation indexes: INR 2.5, APTT 74.6 s, fibrinogen 0.6 g/L. | Arterial-phase contrast-enhanced CT revealed a pancreatic head pseudoaneurysm (gastroduodenal artery branch) and a tiny pre-existing splenic hilum pseudoaneurysm (splenic arterial branch), with duodenal contrast extravasation and peripancreatic hematoma. | Aggressive fluid resuscitation and norepinephrine infusion to maintain blood pressure. Blood transfusion therapy: 6 units of packed red blood cells, 800 mL fresh frozen plasma, and 20 units of cryoprecipitate. Emergency transarterial embolization was performed to occlude the feeding artery of the pancreatic head pseudoaneurysm. | Gastrointestinal bleeding ceased. |
| 4 April 2026 | Abdominal pain and distension with persistent peritoneal irritation signs. Vital signs: heart rate 90 beats/min, blood pressure 124/98 mmHg. | Hemoglobin 68 g/L, lactate 2.9 mmol/L, CRP 62 mg/L, procalcitonin 5.49 ng/mL. Coagulation indexes: INR 1.58, APTT 33.6 s, fibrinogen 1.43 g/L. | Non-contrast abdominal CT revealed new high-density free intra-abdominal fluid, consistent with acute recurrent intra-abdominal hemorrhage. | Continued symptomatic supportive treatment. Blood transfusion therapy: 2 units of packed red blood cells, 200 mL fresh frozen plasma, and 10 units of cryoprecipitate. Second Emergency transarterial embolization was performed to occlude the residual feeding artery of the pancreatic head pseudoaneurysm. | Vital signs remained stable without active bleeding |
| 14 April 2026 | Progressive aggravated abdominal distension and abdominal pain, recurrent massive intra-abdominal hemorrhage. Vital signs: heart rate 112 beats/min, blood pressure 92/60 mmHg, hemodynamic instability. | Hemoglobin 62 g/L, lactate 5.7 mmol/L, CRP 75 mg/L. Coagulation indexes: INR 2.01, APTT 41.2 s, fibrinogen 1.02 g/L. | Arterial-phase contrast-enhanced abdominal CT demonstrated marked interval enlargement of the pre-existing splenic hilar pseudoaneurysm, with extensive peripheral hemorrhagic exudation. | Aggressive fluid resuscitation and high-dose norepinephrine infusion (cumulative dose: 24 mg) were administered. Comprehensive supportive and anti-infective treatment was continued. Repeated blood product transfusion (6 units packed red blood cells + 400 mL fresh frozen plasma) was performed. | Uncontrolled massive hemorrhage and multiple organ dysfunction, with eventual clinical death. |
